# The Family Level Assessment of Screen Use–Mobile Approach: Development of an Approach to Measure Children’s Mobile Device Use

**DOI:** 10.2196/40452

**Published:** 2022-10-21

**Authors:** Oriana Perez, Anil Kumar Vadathya, Alicia Beltran, R Matthew Barnett, Olivia Hindera, Tatyana Garza, Salma M Musaad, Tom Baranowski, Sheryl O Hughes, Jason A Mendoza, Ashutosh Sabharwal, Ashok Veeraraghavan, Teresia M O'Connor

**Affiliations:** 1 United States Department of Agriculture/Agricultural Research Service Children's Nutrition Research Center Baylor College of Medicine Houston, TX United States; 2 Department of Electrical & Computer Engineering Rice University Houston, TX United States; 3 Center for Research Computing Rice University Houston, TX United States; 4 Baylor College of Medicine Houston, TX United States; 5 Public Health Sciences Division Fred Hutchinson Cancer Center Seattle, WA United States; 6 Center for Child Health, Behavior and Development Seattle Children's Research Institute Seattle, WA United States

**Keywords:** screen time, mobile media apps, children, mobile phone use, tablet use, mobile phone

## Abstract

**Background:**

There is a strong association between increased mobile device use and worse dietary habits, worse sleep outcomes, and poor academic performance in children. Self-report or parent-proxy report of children’s screen time has been the most common method of measuring screen time, which may be imprecise or biased.

**Objective:**

The objective of this study was to assess the feasibility of measuring the screen time of children on mobile devices using the Family Level Assessment of Screen Use (FLASH)–mobile approach, an innovative method that leverages the existing features of the Android platform.

**Methods:**

This pilot study consisted of 2 laboratory-based observational feasibility studies and 2 home-based feasibility studies in the United States. A total of 48 parent-child dyads consisting of a parent and child aged 6 to 11 years participated in the pilot study. The children had to have their own or shared Android device. The laboratory-based studies included a standardized series of tasks while using the mobile device or watching television, which were video recorded. Video recordings were coded by staff for a gold standard comparison. The home-based studies instructed the parent-child dyads to use their mobile device as they typically use it over 3 days. Parents received a copy of the use logs at the end of the study and completed an exit interview in which they were asked to review their logs and share their perceptions and suggestions for the improvement of the FLASH-mobile approach.

**Results:**

The final version of the FLASH-mobile approach resulted in user identification compliance rates of >90% for smartphones and >80% for tablets. For laboratory-based studies, a mean agreement of 73.6% (SD 16.15%) was achieved compared with the gold standard (human coding of video recordings) in capturing the target child’s mobile use. Qualitative feedback from parents and children revealed that parents found the FLASH-mobile approach useful for tracking how much time their child spends using the mobile device as well as tracking the apps they used. Some parents revealed concerns over privacy and provided suggestions for improving the FLASH-mobile approach.

**Conclusions:**

The FLASH-mobile approach offers an important new research approach to measure children’s use of mobile devices more accurately across several days, even when the child shares the device with other family members. With additional enhancement and validation studies, this approach can significantly advance the measurement of mobile device use among young children.

## Introduction

### Background

Screen use includes mobile device use, television watching, playing video games, and computer use. Strong associations have been demonstrated between increased screen time and worse sleep outcomes, locomotive skills, and dietary habits in children [1-4]. Screen-based activities, particularly with television and video games, may be related to poor academic performance, whereas mobile phone use has been shown to be correlated with poor mental health in adolescents [5-7]. Screen media use has also been associated with less favorable body composition and worse cardiometabolic risk in youth [1,8], but the associations seem less clear in longitudinal studies [9]. Limitations in measurement might explain discrepancies in the association of screen use with health outcomes in the literature, raising concern over the accuracy of screen media exposure assessments in past studies.

The most common approach to measuring screen use is through self-report or parent-proxy report of children’s screen use by questionnaires, which introduces errors and biases [10], affecting the conclusions drawn from the studies. Within this context, screen time has continued to rise among children and adolescents and increasingly includes the use of mobile devices, such as tablets and smartphones [11,12]. Approximately, 48% of children aged 0 to 8 years personally owned a mobile device in 2020 [12], and 41% of tweens (aged 8-12 years) and 84% of teens had their own smartphone in 2019 [11]. This increased variety of screen use among youth supports the need for accurate measurements of different types of screen use to inform outcome-based research studies. The gold standard for measuring children’s television viewing remains to be direct observation [10], but its use is limited by expense and privacy concerns. Observation can be even more challenging when used to measure mobile screen use. Parent-proxy–reported general estimates are less cumbersome and less costly, but parent report of young children’s mobile device use had a high frequency of underestimation and overestimation of use compared with objective assessment (35.7% and 34.8%, respectively) [13]. Mobile screen use by children may be particularly difficult to assess because of its intermittent use, the short duration of the use of some apps [13], and the use of devices away from parents because of the mobile nature of the device.

The difficulties in accurately measuring children’s mobile device use demonstrate an acute need for new measurement tools to accomplish this goal. We have already developed a novel approach for measuring children’s television viewing [14]. There are also assessment tools to track the screen use of a person on a mobile device used only by that person [13,15,16]. However, the existing methods cannot distinguish the user of the device, which is an important consideration for children who may access a parent’s phone or share a device with a sibling.

### Objectives

In this study, our goal was to develop an innovative approach that leverages the capabilities of smartphone apps to develop a less intrusive yet accurate method for assessing children’s mobile screen use. This study describes the Family Level Assessment of Screen use (FLASH) for mobile devices. The FLASH-mobile approach makes use of (1) an app to track device and app use logging, (2) notifications to identify the user of the device, (3) a system to provide feedback on compliance with the user identification notification to improve compliance, and (4) a minimum threshold of user identification to be included in the analysis. In this study, we used the HealthSense platform [17] for implementing the FLASH-mobile approach with device and app use tracking and user identification, but the FLASH-mobile approach could be implemented with other Android tracking apps in the future. This study describes the development of and assesses the feasibility of the FLASH-mobile approach to track children’s mobile smartphone or digital tablet use, compliance with the user log-in step, and parents’ perceptions of the FLASH-mobile approach.

## Methods

### Overview

A total of 4 feasibility studies tested multiple iterations of the FLASH-mobile approach on mobile phones and tablets. Participants included a parent and at least one child aged 6 to 11 years. Data were collected from December 2019 to August 2021 by installing HealthSense, an app that tracks the use of the device and corresponding apps, on the participants’ mobile phone or tablet.

### Ethical Considerations

This research was approved by the Baylor College of Medicine Institutional Review Board (H-40556), with a reciprocal authorized agreement with Rice University. Parents provided written, informed consent for themselves and their child, and the children provided assent to participate.

### Participants

Participants were recruited from the community and had to live in the United States, speak English, and be a parent of a child aged between 6 and 11 years, and the child had to have their own or share another family member’s Android device (smartphone or tablet). Participants were excluded if the parent or child had a developmental, medical, mental, or physical condition that would prevent them from following the study protocol. Families with mobile devices with other operating systems (OSs), such as Apple iOS or Amazon Fire OS, were excluded because other OSs do not support the HealthSense platform. For laboratory-based protocols, a study-owned backup device was available in case of technical difficulties with the participant’s device. Families were compensated US $40 for their participation.

### FLASH-Mobile Approach

The FLASH-mobile approach was developed to estimate the total time a target child spent using a mobile device. Children often share a mobile device with their siblings or a parent. Therefore, 4 steps were developed to assess the mobile device use of a child from a shared device: (1) an app to track the Android OS app use statistics on the device to identify mobile device use time, duration, and which apps were used; (2) identification of the user of the mobile device via device notifications when unlocked; (3) a system to provide feedback on compliance with the user identification notifications to improve compliance; and (4) a minimum threshold of user identification compliance to be included in the analysis. Several iterative versions of the user identification procedure were tested based on feedback from the participants on earlier versions.

The FLASH-mobile approach was implemented using the HealthSense app [17], developed by researchers at Rice University and used for all the validation and testing results presented in this paper. Similar to other apps such as Chronicle [13] and Minuku [18], HealthSense uses Android’s UsageStatsManager [19] function to access app use event entries recorded by the phone’s OS [20]. Specifically, each time an app is opened (moved to foreground) or closed (moved to background), the Android OS automatically records these as individual time-stamped events in the Android event log, regardless of whether HealthSense or any other app is installed on the device. HealthSense reads this time-stamped event log, resulting in accurate app use data. To monitor phone unlock events for user identification, the app monitors another automatic event log, the ACTION_USER_PRESENT [21] event. When the participants installed the HealthSense app, they were explicitly asked to authorize the app to read these event logs that the Android OS had already recorded on their devices.

For the user identification step, multiple approaches were tested: version 1 (V1), a pop-up prompt appeared when the phone was unlocked, which had to be answered to use the device; version 2 (V2), a notification banner appeared when the phone was unlocked and after every 15 minutes of use; and version 3 (V3), a notification banner that appeared only when the phone was unlocked. In each case, the user had to identify themselves by selecting 1 of 2 options, an orange button with the word *child* or a gray button with the word *other* (Multimedia Appendix 1). There was no option for multiple users. To refine the log-in prompt, three major requirements were considered: (1) the app should be compatible across as many Android versions as possible, (2) newer versions of the Android OS had to support it, and (3) the log-in prompt must be user-friendly to encourage high compliance among children.

We conducted 4 feasibility studies. Parents were instructed to download the HealthSense app. The type of Android device used, model number of the device, OS number, whether the HealthSense app was successfully downloaded, and whether the user identification feature worked as expected were tracked. Two laboratory-based protocols (Table 1: feasibility studies A [in laboratory] and B [in laboratory]) assessed the ability of the FLASH-mobile approach to accurately capture participants’ mobile device use, compared with the research staff’s assessment of the participants’ gaze on their mobile device from video data. Parents and children were observed in a laboratory set up like a living room while being video recorded by 4 high-definition cameras placed in each corner. The protocols included a series of tasks (eg, using the mobile phone or tablet, watching television, playing with the toys in the room, or free play).

Furthermore, 2 home-based studies (Table 1: feasibility studies C [at home] and D [at home]) gathered real-world information about the ability of the FLASH-mobile approach to collect data on the child’s smartphone (study C [at home]) and tablet use (study D [at home]) over 3 days. Feasibility was assessed by the (1) number of mobile devices the HealthSense app could be downloaded on for the at-home and laboratory studies, (2) ability of the app to collect data over the 3-day at-home study period, (3) compliance with user notification, and (4) parents’ perceptions of the FLASH-mobile approach during the exit interviews. All 3 versions of HealthSense (V1, V2, and V3) were iteratively tested in study C (at home). We observed low participant compliance with V3, which prompted a modification of the protocol to include feedback for compliance. Feedback on compliance was accomplished by sending reminders to respond to the user notification via telephone, SMS text message, or email to participants with low compliance and by sending encouraging messages to participants with high compliance.

**Table 1:** Description of the feasibility studies (N=48).

Feasibility study	Setting	HealthSense app version tested	Parent-child dyads enrolled, n (%)	Device tested	Duration
A	Laboratory based	Version 1^a^	5 (10)	Mobile phone	Approximately 2 hours (video-recorded tasks)
B	Laboratory based	Version 3^b^	10 (21)	Mobile phone or tablet	Approximately 2 hours (video-recorded tasks)
C	Home based	Versions 1, 2^c^, and 3	21 (44)	Mobile phone	3 days (naturalistic)
D	Home based	Version 3	12 (25)	Tablet	3 days (naturalistic)

^a^A pop-up prompt appeared when the device was unlocked, and the user had to identify themselves by selecting 1 of 2 options, *child* or *other*, to use the device. It is no longer supported by the Android operating system.

^b^A notification banner appeared only when the phone was unlocked, and the user had to identify themselves by selecting 1 of 2 options, *child* or *other*. Daily text or email feedback was sent to the family on their compliance.

^c^The notification banner appeared when the phone was unlocked and after every 15 minutes of use. The user had to identify themselves by selecting 1 of 2 options, *child* or *other*.

### Video Coding

Screen use was defined as the duration of time a child spent actively watching the mobile device screen (gaze on the mobile device) or using the screen while multitasking (eg, while playing with toys). This is similar to how others have defined screen use, including as background television [22]. A total of 5 research staff members coded video data from studies A (in laboratory) and B (in laboratory) to identify when the target child was using or watching the mobile device during the observation protocol to provide a gold standard for assessing the accuracy of the FLASH-mobile approach in tracking child device use. The 4 video frames were viewed simultaneously by staff to get 4 different angles of view of the room during coding. The video was coded using duration coding with one of ten codes: (1) child screen use, (2) parent screen use, (3) both screen use, (4) child multitasking, (5) child audio use, (6) parent audio use, (7) both audio use, (8) no mobile device use, (9) uncertain, or (10) out of frame (informed by previous publications [23-25]). Screen use was differentiated from audio use based on whether the person was gazing at the screen or making a call, speaking voice commands, or listening to music. To be considered screen use, gaze could be quickly diverted for <3 seconds but should still mainly be focused on the mobile device, similar to other coding protocols [26]. The *multitasking**** ****code* was applied when the child used a mobile device screen concurrently with other tasks, such as watching television, playing with toys, or talking to another person [24]. In all, 10% of each video was double coded by 2 staff members, and the agreement was high [27-29] (feasibility study A [in laboratory], Cohen κ=0.76, SD 0.38; feasibility study B [in laboratory], Cohen κ=0.79, SD 0.37).

### Identification of the Apps Used

HealthSense listed the names of all the opened apps. A log of the apps used was generated when the child was the user. These were categorized by the type of app: educational (eg, Math Jumps and Khan Academy Kids), streaming video services (eg, YouTube and Netflix), gaming, social media, browsing, Android OS, and others (Multimedia Appendix 2 presents the full list of categorized apps). Two staff members reviewed and independently coded the apps into one of these categories by reading the description included in the Google App Store. A third staff member reviewed the app categorization. Differences in the categorization of apps were reviewed and discussed as a team until a consensus was reached.

### Exit Interviews

All the parents were asked to share their perceptions of using the FLASH-mobile approach and provide suggestions for its improvement. The semistructured interviews followed a standardized script, which included probes and prompts to clarify responses (Multimedia Appendix 3 presents the full interview script). For studies C (at home) and D (at home), parents and children were provided with a copy of their mobile device use over the past 3 days before their interview and were asked to review it. The log included which apps were used, the time of day, the duration of use, and the user identified (*child*, *other*, or none if the user identification prompt was ignored). Parents were asked to verify whether the apps listed were correct; whether any apps were missing or added incorrectly; and whether the timing, duration, and user identified were correct. All the interviews were audio recorded, transcribed, and coded by 3 study staff members (OH, TG, and OP), using NVivo (version 11, QSR International) software. A codebook was created based on an inductive coding of themes [30,31]. Pairs of reviewers independently coded each interview. Discrepancies in the themes were discussed and resolved until a consensus was reached. The authors identified the main themes which arose and categorized the subthemes within them.

### Analyses

Means, SDs, and percentages describe the demographics and dyad’s compliance with user identification. User compliance was measured along 2 dimensions: compliance with the log-in identification and unidentified use. Compliance score was defined as the percentage of times the user responded to the log-in notification over the total number of times the mobile device was unlocked. Unidentified use was calculated as the percentage of use for which the user was not known over the total mobile device use.

*Child use* of device was defined by collating staff video codes of (1) child directly using mobile screen alone, (2) using mobile screen with someone else, and (3) multitasking mobile screen use with other activities (eg, playing). The agreement between the HealthSense output and staff coding for mobile screen use in feasibility studies A (in laboratory) and B (in laboratory) was tested using the percentage of agreement, prevalence- and bias-adjusted Cohen κ statistic, and intraclass correlation coefficient.

## Results

### Participant Demographics

A total of 48 parent-child dyads participated in the study (n=5, 10% for study A [in laboratory]; n=10, 21% for study B [in laboratory]; n=21, 44% for study C [at home]; and n=12, 25% for study D [at home]). Each dyad participated in only 1 study. Of all the 48 enrolled participants, 33 (68%) were able to download the app on their own device and have all the components function. For the observational laboratory studies, a study device was available for 2 families whose device did not work, resulting in 73% (35/48) of participants with complete data from the HealthSense app. The demographics of the sample are shown in Table 2, and those for each study can be found in Multimedia Appendix 4.

**Table 2:** Demographic characteristics of participants with complete data (N=48).

Characteristic			All tests with incomplete data	All tests with complete data
**Target children, n (%)**			13 (27)	35 (73)
	Age (years), mean (SD)		8.6 (1.9)	8.4 (1.5)
	Sex (female), n (%)		6 (46)	18 (51)
	**Race or ethnicity, n (%)**			
		Non-Hispanic White	3 (23)	8 (23)
		Hispanic White	3 (23)	10 (28)
		Non-Hispanic Black	5 (39)	9 (26)
		Hispanic Black	0 (0)	1 (3)
		Asian	0 (0)	2 (6)
		Hispanic, non-Hispanic mix, other, and unknown	2 (15)	5 (14)
**Parent, n (%)**			13 (27)	35 (73)
	Age (years), mean (SD)		40.1 (4)	38.3 (6)
	Sex (female), n (%)		13 (100)	33 (94)
	**Race or ethnicity, n (%)**			
		Non-Hispanic White	2 (18)	10 (28)
		Hispanic White	3 (25)	11 (31)
		Non-Hispanic Black	5 (42)	9 (26)
		Asian	1 (8)	2 (6)
		Hispanic, non-Hispanic mix, other, and unknown	1 (8)	3 (8)
	**Education, n (%)**			
		High school graduate	1 (8)	1 (3)
		Technical school	1 (8)	4 (11)
		Some college	3 (25)	12 (34)
		College	4 (33)	9 (26)
		Graduate school	3 (25)	9 (26)
	**Income (US $), n (%)**			
		<30,000	0 (0)	6 (17)
		<60,000	7 (58)	10 (28)
		>60,000	5 (42)	18 (51)
		Do not know	0 (0)	1 (3)

### HealthSense Versions Tested and Installation Issues

Problems with the installation of the app on participants’ mobile devices included the nonappearance of user identification, lack of tracking app use, inability to install HealthSense on the device, problems due to phones and tablets with older Android OS systems (<8), and incompatibility of certain mobile device models with the HealthSense app. In some cases, the reason for malfunction was unknown. A full description of installation issues is provided in Multimedia Appendix 5.

### Usability of the HealthSense App Interface and Participant Compliance

**Figure 1:**
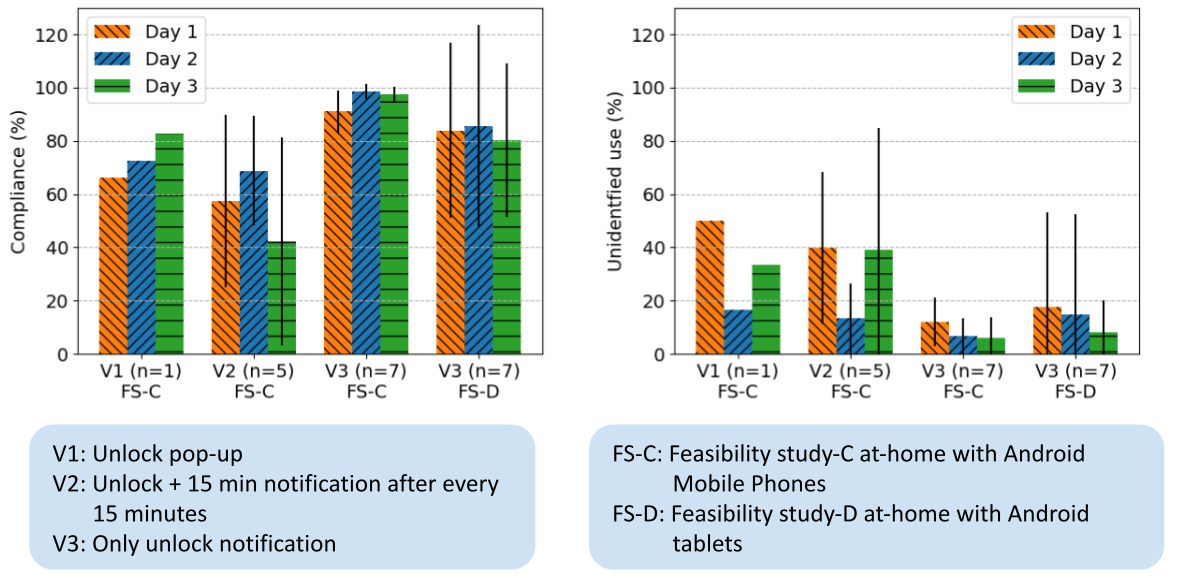
Compliance of user identification and percentage of unidentified mobile device use across the different versions of HealthSense. V1: version 1; V2: version 2; V3: version 3.

Compliance with the user identification procedure for the at-home feasibility tests increased over the iterations of the HealthSense app from versions V1 to V3 (Figure 1). HealthSense V3, which included reminders and encouraging messages sent to participants daily, achieved the highest compliance among users, with 95.7% compliance among smartphone users and 83.3% for tablet users maintained across 3 days of the feasibility study (Figure 1, left graph). Figure 1 also illustrates the percentage of unidentified use in the total mobile device use, which decreased as the compliance approached 100% (Figure 1, right graph). Note that the percentage of unidentified use time could be high even with relatively high compliance, for instance, if someone used the mobile device for a long time without identifying themselves at the start of that use period.

### Accuracy of the FLASH-Mobile Approach Compared With Gold Standard

The accuracy of the FLASH-mobile approach in capturing children’s mobile device use was assessed in the laboratory observation, and it was found that most users (12/15, 80%) had high compliance with the log-in notification, with >80% of use identified, whereas a smaller proportion (3/15, 20%) had very low compliance, with >70% of use unidentified. When the user compliance was low, HealthSense identified app use but could not identify who used the app, resulting in an unidentified use label and disagreement with staff coding between *child* or *other*’s use. Hence, a threshold of ≥70% of identified use in the overall use of the mobile device was selected to identify quality assessments for reporting target child’s mobile device use. Those with lower identified mobile device use were screened out because their inclusion would lead to discrepancies resulting from user behavior (low compliance) rather than HealthSense app behavior.

For the users with high compliance (≥70% of app use identified), HealthSense identified the target child’s mean use as 21.76 (SD 20.71) minutes, whereas the gold standard was 18.30 (SD 10.77) minutes. Among the 12 families with high compliance with user identification, HealthSense achieved a 73.6% (range 44.7%-96.14%) mean agreement with human labelers in capturing the target child’s mobile use, 78.9% (range 3.55%-97.5%) in capturing other’s mobile use, 2.9% (range 0%-17.75%) agreement in capturing unidentified mobile use, and 85.2% (range 38.37%-98.37%) agreement in capturing no mobile use. Among the 3 families with low compliance (<70% of app use identified), mean agreement between human labelers and target child’s mobile use was 37% (range 0%-78.29%), 2.1% (range 0%-6.33%) agreement in capturing other’s mobile use, 82.7% (range 73.6%-97.17%) agreement in capturing unidentified mobile use, and 95.9% (range 93.82%-98.33%) agreement in capturing no mobile use. The prevalence- and bias-adjusted Cohen κ value for the agreement between HealthSense and the gold standard was 0.711 (SD 0.301), and the intraclass correlation coefficient was 0.714 for the high-compliance users, indicating high agreement between the gold standard and HealthSense estimate [32-35].

The times HealthSense and gold standard disagreed were primarily due to HealthSense either identifying child use as *other* use or no user was identified. The former happened when either parent or sibling logged in as the user and the child started using the device or the target child started coviewing the mobile device along with their parent or sibling, which staff labeled as *child* use. The latter case happened when the user of the device ignored the log-in prompt, failing to identify the user.

### FLASH-Mobile Approach in Naturalistic Setting

After qualitatively reviewing the 3-day app and user log, among 23, only 2 (9%) parents reported minor discrepancies in the mobile use reports of their child (one noted a misidentified user for an app use during the 3-day protocol, and another found 2 apps that they did not recall their child using). The remaining 91% (21/23) of parents agreed with the HealthSense log of their child’s mobile device use.

**Figure 2:**
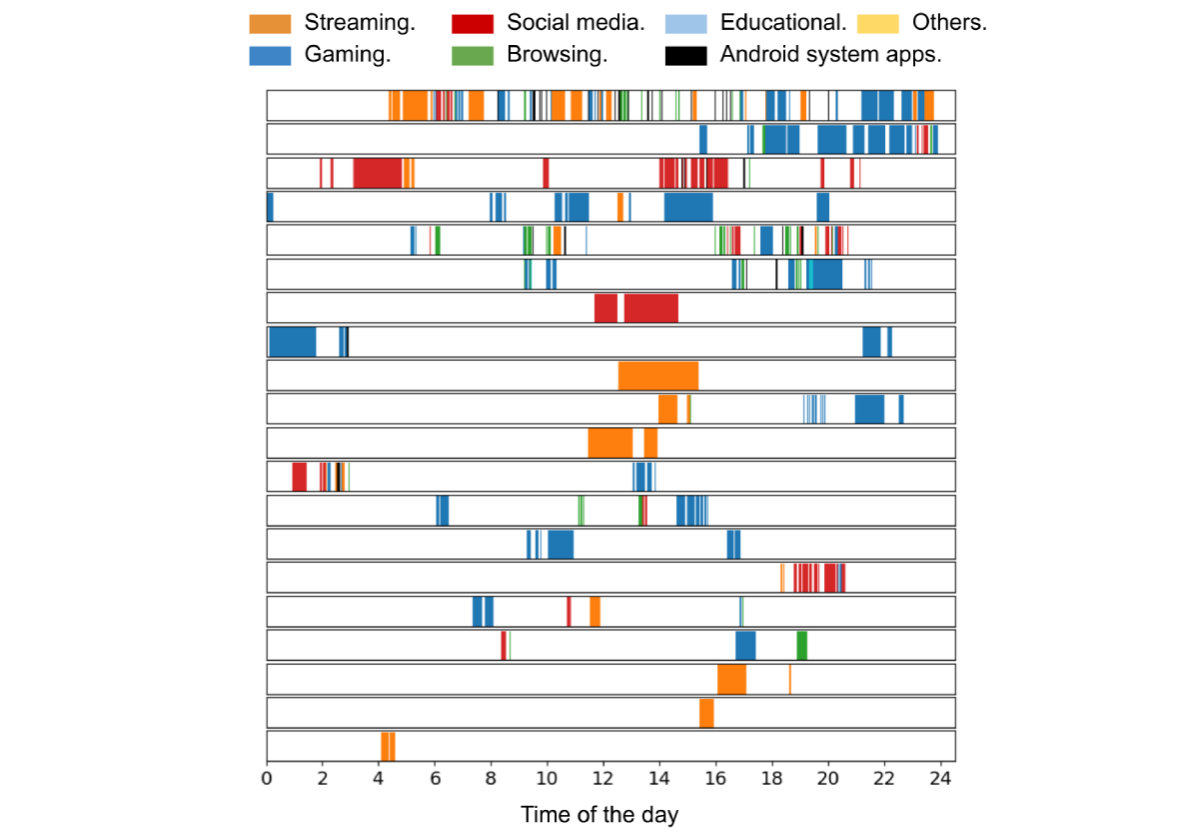
Mobile device app use by 20 children on day 2 of the 3-day at-home study. This included 20 children from feasibility studies C and D. One child in the full sample (N=21) had no mobile use on day 2 and was, therefore, not depicted.

Figure 2 demonstrates the categories of app use of 20 children from the at-home studies on the second day of the 3-day study; a child (not depicted) had no mobile device use on the second day. The children’s device use duration varied widely, ranging from just half an hour per day to >10 hours a day, with 3 to 7 different apps used by the children during a single day.

### Qualitative Feedback From Parents and Children in Exit Interviews

#### Laboratory-Based Studies

The parents from the observation laboratory studies tended to have positive or neutral feelings toward the FLASH-mobile approach, stating that it seemed fine, did not impact the child’s enjoyment while using the phone, and could allow them to track their child’s screen time. Although most parents had no issues with HealthSense and did not think that it was intrusive, there were a few concerns about privacy, for instance, regarding who would have access to the data and what was actually being recorded.

#### At-Home Studies

Most (18/23, 78%) parents from the at-home studies found the app useful for determining how much time their child spent using the mobile device and to review the apps they used. More than half (5/8, 63%) of participants who tested the versions with a user identification pop-up and 15-minute reminders (V1 and V2) disliked the pop-up button. They found the pop-up annoying and too frequent. Those who tested V3, with an unlock user identification notification, had mixed reactions. Approximately, half (9/15, 60%) of the participants liked the notification and found no issues with it, but 13% (2/15) of participants stated that the notification was annoying. The remaining parents (4/15, 27%) had a positive reaction to the notification but added that it was difficult to notice. Moreover, 13% (2/15) of participants reported that the app slowed down their phones and did not allow some calls to come through.

Suggestions for the improvement of the FLASH-mobile approach on phones included simplifying the user identification feature; making it available for other devices such as tablets, televisions, and Apple iOS devices; adding a time-restriction function; adding games or an educational component; having a unique personal identification number or password per user; and blocking phone use until user identification is selected. Others suggested including the user identification requirement only on shared devices. A few parents using mobile phones expressed privacy concerns over their information being sold or stolen and other security risks, and some felt uncomfortable with HealthSense recording not just the child’s but also the parent’s mobile phone use. None of the parents using tablets expressed similar concerns about privacy.

## Discussion

### Principal Findings

The FLASH-mobile approach, a mobile device tracking app on an Android device, included four features: (1) user identification prompts, (2) app use tracking ability, (3) feedback on compliance with the user identification notification via text or email, and (4) a minimum threshold of user identification compliance to best capture mobile device use by a child. This approach was found to be feasible to measure children’s mobile device use. The FLASH-mobile approach addresses differentiating users of shared devices by sending a notification banner for the user to identify themselves every time the mobile device is unlocked along with intermittent communication with participants to praise or remind participants to be compliant. This provided >90% compliance rates with user identification for smartphones and >80% for tablets. In this small feasibility study, the threshold was 70% compliance with user identification, but further studies need to verify whether this threshold continues to correspond to high agreement between the FLASH-mobile approach and gold standard observation. When compliance with user identification reached this threshold, FLASH-mobile approach achieved a 73.6% (SD 16.15%) mean agreement with human labelers in capturing the target child’s mobile use (defined as a child actively watching the mobile device’s screen alone or with someone else or multitasking with mobile device use and other activities such as playing with toys). There is no agreed-upon definition in the literature for screen use by children. Others have conceptualized screen use of mobile devices as any time a device is used by its owner, regardless of whether the person is viewing the screen [13,36]. The latter definition is difficult to apply to a situation where a phone is shared between a child and another user. However, the definition used for screen use by a study will directly affect the agreement of a tool like FLASH-mobile for actually measuring a child’s screen use.

During the course of this study, modifications were made to the FLASH-mobile approach to optimize it and adapt to the continually changing aspects of the Android OS. For example, the full-screen pop-up prompts used in the initial version (V1) of the FLASH-mobile approach are no longer supported by the latest Android OS versions because of new security constraints. Therefore, notification banners were used in the second version (V2), which appeared when the phone was unlocked. In addition, to better identify the user of the device, notification banners were programmed to appear after every 15 minutes of device use. The results from study B (at home) demonstrated that the 15-minute notification was not user-friendly, thus reducing user compliance. Therefore, the final version (V3) of the FLASH-mobile approach included only the unlock notification banner. Such a notification banner is supported by past and current versions of the Android OS. Although a few parents disliked the notification banner, it was well received by most parents and resulted in the lowest proportion of unidentified mobile device use.

Although FLASH-mobile is not publicly available, it could be reproduced using specific Android methods [19-21]. Moreover, similar apps have been developed by others [13,18] and are available for researchers to log device use. The specific features of FLASH-mobile such as user identification notification are straightforward to replicate in any of these Android apps using the methods mentioned [21].

### Comparisons With Prior Work

Only a few other digital tracking approaches have been used to measure children’s mobile device use, such as similar tracking apps on Android devices [13], screenshots of the device’s battery page in the settings on Apple devices [13], or frequent screenshots of the device [16]. However, all these approaches pose challenges in differentiating a child’s use from use by others when the mobile device is shared, which is common among younger children [13]. Only one of these studies assessed children’s mobile device use using an approach similar to FLASH-mobile [13]. Radesky et al [13] examined the association between young children’s mobile device use and their emotion regulation and executive functioning. Parents who had an Android mobile device (n=126) were instructed to download Chronicle [37], an app similar to HealthSense, for a 9-day study period. Parents with an iPhone (n=220) were asked to take screenshots of the device’s battery page in the settings to visualize app use for the past 7 to 10 days. For 71% of the young children who shared the device with someone else, parents completed a form to indicate which apps their child used during the study week. Complete mobile device data were available for 71% of the children, similar to the 69% (33/48) of the participants who could download the HealthSense app with full functionality for the FLASH-mobile approach described here. An important limitation that Radesky et al [13] identified was that they could not identify the user among the 70.6% of the sample who shared the device with someone else; an important issue that the FLASH-mobile approach overcame with the user notification banner. The observational studies (studies A [in laboratory] and B [in laboratory]) for the FLASH-mobile approach additionally demonstrated that when a parent and child were together and used the mobile device, some of that time was shared viewing time. However, most of that occurred appropriately when the child was logged in as the user, ensuring that the use was logged under the child. The duration of time when the parent and child viewed the mobile device together during the observational studies may be artificially high, given that the protocol was performed when the parent and child were together and specifically focused on the screen. It is likely that in free-living conditions, this happens less frequently.

Similar to Radesky et al [13], the FLASH-mobile approach could not be used on all the Android devices tested, and tablets experienced slightly more problems than smartphones. As illustrated in Multimedia Appendix 5, some phones and tablets with older Android OS systems and certain mobile device models did not support the HealthSense app. Inclusion criteria for future studies using background apps that track device and app use should include the use of an Android OS that is newer (≥7.0), and pretests should be conducted to find which specific Android devices support the tracking app. Future studies also need to further explore the appropriate threshold of compliance with user identification that results in valid and reliable data and how many days of mobile device use are required to capture typical mobile device use by a child. The assessment of physical activity using wearable monitors has adopted similar approaches to develop guidelines for scoring and processing accelerometer data with minimum thresholds of hours and days of wear to be considered valid [38].

### Limitations

This study has several important limitations. The sample was small, recruited through convenience sampling, and thus not representative. However, the sample does reflect a racially and economically diverse group, which is important for demonstrating usefulness for studies targeting diverse children. The small sample with a limited number of days of data collection (3 days) was also not meant to establish typical mobile device use among children. Because the FLASH-mobile approach requires an app, such as HealthSense, Chronicle [37], or Minuku [18], that reads the Android’s UsageStatsManager [19] function to track device and app use, it only worked on the Android OS. This may limit the population that can be assessed using this approach. The FLASH-mobile approach also did not work on all Android devices or with all versions of the Android OS. It is important to proactively design studies to screen for mobile devices that support the FLASH-mobile approach. Although the current version of the FLASH-mobile approach leveraged the HealthSense platform for data management, future versions of FLASH-mobile will need to use alternate platforms because the HealthSense platform will be discontinued in the near future. We are already piloting a version of FLASH-mobile using the Chronicle platform [37].

The low compliance of some participants with the log-in notifications was a challenge, as it resulted in high unidentified app use on the mobile device in earlier versions of testing. In the future, user identification prompts should be made more child-friendly, or they may be turned off when the child does not share the mobile device with others. In addition, future versions of the approach could include automated reminders and encouraging messages for participants to comply with user identification instead of relying on staff. We also found that having a uniform auto screen lock time (eg, 2 minutes) increased the app's ability to accurately identify the user (data not presented). However, the problem of user identification arises only when the mobile device is shared. Many young children now have their own mobile device, including 46% of children aged 2 to 4 years and 67% of children aged 5 to 8 years [12], in which case, apps that read the Android’s UsageStatsManager [19] function to track device and app use could more easily track the child’s mobile device use.

### Conclusions

The FLASH-mobile approach can be implemented using a mobile device app on an Android device with 4 features: user identification prompts, device and app use tracking ability, feedback on compliance with user identification, and thresholds of compliance to identify valid data. It offers an important new research approach to more accurately measure children’s use of mobile devices across one or several days, even when the child shares the device with others. By providing time-stamped data of app use for a specific user, the FLASH-mobile approach will allow for better causal assessments of how the duration, timing, and type of mobile device use by children can affect their developmental and health outcomes. Other researchers can use this approach to further advance the measurement of mobile device use among children.

## Appendix

Multimedia Appendix 1 Mobile app interface.

Multimedia Appendix 2 Classification of apps.

Multimedia Appendix 3 Interview script.

Multimedia Appendix 4 Demographic characteristics of participants with complete and incomplete data.

Multimedia Appendix 5 HealthSense versions tested and problems during study.

## Abbreviations

FLASH: Family Level Assessment of Screen Use
OS: operating system
V1: version 1
V2: version 2
V3: version 3
