# Supplementary material for: The Family Level Assessment of Screen Use–Mobile Approach: Development of an Approach to Measure Children’s Mobile Device Use
Source: JMIR Form Res. 2022 Oct 21;6(10):e40452. doi: 10.2196/40452 (PMC9636534; doi:10.2196/40452)
Supplement: Multimedia Appendix 1 [file formative_v6i10e40452_app1.docx]

**Appendix 1 – Mobile app interface**


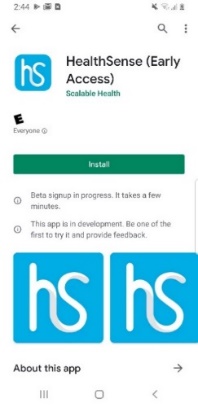


Fig 1. Healthsense app store page


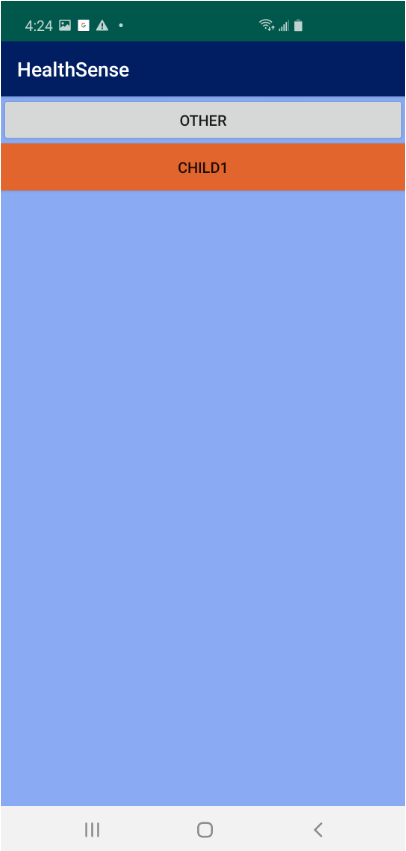

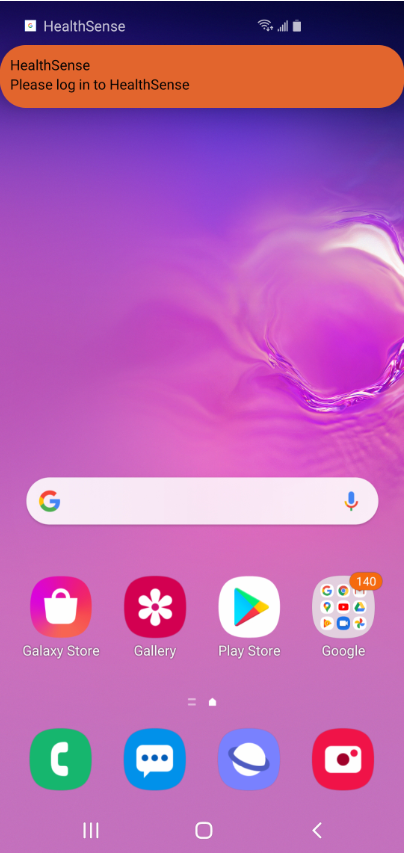


Fig 2. User identification prompt and buttons for participants
