# Supplementary material for: The Family Level Assessment of Screen Use–Mobile Approach: Development of an Approach to Measure Children’s Mobile Device Use
Source: JMIR Form Res. 2022 Oct 21;6(10):e40452. doi: 10.2196/40452 (PMC9636534; doi:10.2196/40452)
Supplement: Multimedia Appendix 2 [file formative_v6i10e40452_app2.docx]

**Appendix 2. Classification of apps**

| **Social Media/Messaging** | Facebook, groupme, Instagram, like, snapchat, zhiliaoapp, whatsapp |
| --- | --- |
| **Browsing/Reading** | Calculator, camera, chrome, contacts, dollargeneral, gallery3d, gm, googlequicksearchbox, notes, particlenews.newsbreak, smartnews, vending, weather, horo, sbrowser |
| **Video Streaming** | Odysseyadventureclub, video, ncikjr, Pluto, youtube, disney.disneyplus |
| **Educational** | khankids.android, starfall.ltr, kindle, mathgames.jump |
| **Gaming** | Ibispaintx, topgamesinc, building.Lokicraft, cornhole, drawrider, fruitninjafree, gachalife, geishatokyo, h8games, halfbric, helixjump, imayi.dinosaurparkfree, imayi.oceanexplorer, innersloth.spacemafia lunime, MadOut, minecraftpe, Mojang, pixelcoloring, pou, puzzingopro, roblox, RyansTag, cottgames.fivenightsatfreddys, SkateSpace, snowballio, supercell.brawlstars, WildWorks, candywriter.bitlife, kiloo.subwaysurf, lordsmobile, miniclip.bowmasters, paint, playmobil.reiterhof, pokemongo, uniconstudio.braintest2new, mc, mcpe, word, amanotes, beathopper, tapped, atalogue.global, kiloo.subwaysurf, tachyon, tap2, connect, sport |
| **Android system** | Dialer, incallui launcher, launcher3, systemui, vvm, mms, mdecservice, server.telecom, rocketclean, |
| **Music** | iheartradio |
