# Supplementary material for: The Family Level Assessment of Screen Use–Mobile Approach: Development of an Approach to Measure Children’s Mobile Device Use
Source: JMIR Form Res. 2022 Oct 21;6(10):e40452. doi: 10.2196/40452 (PMC9636534; doi:10.2196/40452)
Supplement: Multimedia Appendix 3 [file formative_v6i10e40452_app3.docx]

**Appendix 3– Interview script**

**FLASH-Mobile Beta Test 4**

**Interview Script**

**Goal of interview:** obtain feedback from parents on the testing of the HealthSense app on your digital tablet for three days.

**Interview may take place in person at CNRC, or over the telephone depending on the participant’s preference.**

**Thank you and your child for agreeing to test the Health Sense App in your** digital tablet**. We want to know what you thoughts are about having this App installed on {your OR your child’s}** digital tablet **for recording your child’s mobile device usage. Your opinion is very important to us, so please be honest and tell us what you really think. There are no right or wrong answers – just what your honest opinion. This will help us to improve the App to make it more user friendly for our participants.**

**I would like to record the interview so that I will not miss any of the important information you share with me. Your name will not be mentioned to protect your confidentiality. Is this ok with you?**

*If yes,* ***Continue Interview***

***If no,*** ***do not record the interview and take notes.***

**Do you have any questions before we begin?**

*If yes****,*** *answer any questions they may have.*

*If no, continue interview*

**All right, let us begin.**

**(START AUDIO RECORDING)**

**Time Start : ____:____am/pm**

**[As soon as you start the interview, state the participant’s Id number, date of the interview and for which study the interview is being completed.]**

1. **What do you think about allowing researchers to use an App like this to track your child’s** digital tablet **use during a specific study period?**

[Probe: to clarify for their responses; ask for examples if they cannot clearly express their ideas]

1. **Did you or your child have any problems with the HealthSense App during the study period? If yes, please describe the problem.**

[Probe: to clarify for their responses; ask for examples if they cannot clearly express their ideas]

1. **Did you or your child have any problems with your** digital tablet **during the study period? If yes, please describe the problem.**

[Probe: to clarify for their responses; ask for examples if they cannot clearly express their ideas]

1. **The HealthSense app displays a notification asking who the user is every time you unlock your device.**

**a. What are your thoughts about the notification that is displayed when the phone is unlocked?**

[Probe: to clarify for their responses; ask for examples if they cannot clearly express their ideas]

**b.**

Prompts:

1. Do you have any suggestions for changes?

**Show parent and child the HealthSense time log report of device usage for the 72 hours.**

1. **In general, do these records match with what you believe to be your child (and your)** digital tablet **usage for the three study days?**
2. Why not/why yes?
3. What do you think of the information on these three days?
4. What, if anything, in this report looks incorrect to you?
5. What, if anything, surprises you about this report?
6. **Now let’s look more carefully at the record for yesterday/Day 3.**

**Do these records match with** digital tablet **usage for {you and child OR for your child} for these 24 hours? Review the specific details with the parent and child.**

1. What, if anything, in this report looks incorrect to you?
2. Are there app use records that you do not believe occurred? If so, please explain.
3. Are there any times you remember you or your child using the device that is not recorded on this report? Please explain.
4. What, if anything, surprises you about this report?
5. **In general, do you have suggestions for how to make the HealthSense app better for other families who take part in studies like this?**

[Probe: to clarify for their responses; ask for examples if they cannot clearly express their ideas]

1. **Do you have any concerns about the HealthSense app?**

[Probe: to clarify for their responses; ask for examples if they cannot clearly express their ideas]

**Before we finish, is there anything else you want to tell me about the app that you have not had an opportunity to say?** [For any comments or suggestions given, probe to ensure a full understanding of what they say].

*STOP RECORDING*

**Time End: ____:____ am/pm**

*Interview Conclusion:*

**Thank you for your time and participation in this interview. I appreciate your talking with me today. Your responses will help us make the app more user friendly.**
