# Supplementary material for: The Family Level Assessment of Screen Use–Mobile Approach: Development of an Approach to Measure Children’s Mobile Device Use
Source: JMIR Form Res. 2022 Oct 21;6(10):e40452. doi: 10.2196/40452 (PMC9636534; doi:10.2196/40452)
Supplement: Multimedia Appendix 4 [file formative_v6i10e40452_app4.docx]

**Appendix 4. Demographic characteristics of participants with complete and incomplete data.**

|  | Overall  Complete and incomplete | All incomplete data | All tests  With complete data | Feasibility  Test A  complete data | Feasibility  Test B  complete data | Feasibility  Test C  complete data | Feasibility  Test D  complete data |
| --- | --- | --- | --- | --- | --- | --- | --- |
| **Children (n)** | 48 | 13 | 35 | 5 | 10 | 13 | 7 |
| Age (years) (mean, SD) | 8.48 (1.557) | 8.62 (1.850) | 8.43 (1.461) | 8.4 (1.14) | 8.40 (1.43) | 8.62 (1.502) | 8.14 (1.864) |
| Sex (% ) |  |  |  |  |  |  |  |
| Female | 24 (50.0) | 6 (46.2) | 18 (51.4) | 2 (40) | 6 (60.0) | 7 (53.8) | 3 (42.9) |
| Male | 24 (50.0) | 7 (53.8) | 17 (48.6) | 3 (60) | 4 (40.0) | 6 (46.2) | 4 (57.1) |
| Race/Ethnicity |  |  |  |  |  |  |  |
| Non-Hispanic White | 11 (22.9) | 3 (23.1) | 8 (22.9) |  | 2 (20.0) | 3 (23.1) | 3 (42.9) |
| Hispanic White | 13 (27.1) | 3 (23.1) | 10 (28.6) | 3 (60) | 3 (30.0) | 3 (23.1) | 1 (14.3) |
| Non-Hispanic black | 14 (29.2) | 5 (38.5) | 9 (25.7) | 2 (40) |  | 6 (46.2) | 1 (14.3) |
| Hispanic Black4 | 1 (2.1) |  | 1 (2.9) |  | 1 (10.0) |  |  |
| Asian | 2 (4.2) |  | 2 (5.7) |  | 2 (20.0) |  | 2 (28.6) |
| Hispanic/Non-Hispanic Mix, Other, Unknown | 7 (14.6) | 2 (15.4) | 5 (14.3) |  | 2 (20.0) | 1 (7.7) |  |
|  |  |  |  |  |  |  |  |
| **Parent (n)** | 48 | 13 | 35 | 5 | 10 | 13 | 7 |
| Parent age (years) (mean, SD) | 38.77 (5.431) | 40.08 (4.425) | 38.29 (5.742) | 39.8 (7.25) | 41.40 (6.096) | 37.38 (4.629) | 34.43 (3.994) |
| Sex (%) |  |  |  |  |  |  |  |
| Female | 46 (95.8) | 13 (100) | 33 (94.3) | 4 (80) | 9 (90.0) | 13 (100) | 7 (100) |
| Male | 2 (4.2) |  | 2 (5.7) | 1 (20.0) | 1 (10.0) |  |  |
| Race/Ethnicity |  |  |  |  |  |  |  |
| Non-Hispanic White | 13 (27.1) | 2 (18.2) | 10 (28.6) |  | 4 (40.0) | 3 (23.1) | 3 (42.9) |
| Hispanic White | 14 (29.2) | 3 (25.0) | 11 (31.4) | 3 (60) | 4 (40.0) | 3 (23.1) | 1 (14.3) |
| Non-Hispanic black | 14 (29.2) | 5 (41.7) | 9 (25.7) | 2 (40) |  | 6 (46.1) | 1 (14.3) |
| Asian | 3 (6.3) | 1 (8.3) | 2 (5.7) |  | 2 (20.0) |  |  |
| Hispanic/Non-Hispanic Mix, Other, Unknown | 4 (8.3) | 1 (8.3) | 3 (8.6) |  |  | 1 (7.7) | 2 (28.6) |
| Education |  |  |  |  |  |  |  |
| HS Graduate | 2 (4.2) | 1 (8.3) | 1 (2.9) |  | 1 (10.0) |  |  |
| Technical School | 5 (10.4) | 1 (8.3) | 4 (11.4) |  |  | 2 (15.4) | 2 (28.6) |
| Some College | 15 (31.3) | 3 (25.0) | 12 (34.3) | 3 (60) | 2 (20.0) | 5 (38.5) | 2 (28.6) |
| College | 14 (29.2) | 4 (33.3) | 9 (25.7) | 1 (20) | 1 (10.0) | 4 (30.8) | 3 (42.9) |
| Graduate School | 12 (25.0) | 3 (25.0) | 9 (25.7) | 1 (20) | 6 (60.0) | 2 (15.4) |  |
| Income |  |  |  |  |  |  |  |
| < $30,000 | 6 (12.5) |  | 6 (17.1) | 1 (20) | 2 (20.0) | 2 (15.4) | 1 (14.3) |
| > $30,000 - < $60,000 | 17 (35.4) | 7 (58.3) | 10 (28.6) | 1 (20) | 1 (10.0) | 5 (38.5) | 3 (42.9) |
| > $60,000 | 24 (50.0) | 5 (41.7) | 18 (51.4) | 3 (60) | 6 (60.0) | 6 (46.2) | 3 (42.9) |
| Do not know | 1 (2.1) |  | 1 (2.9) |  | 1 (10.0) |  |  |
|  |  |  |  |  |  |  |  |

In families where sibling was enrolled, demographics are not reported.
