# Supplementary material for: The Family Level Assessment of Screen Use–Mobile Approach: Development of an Approach to Measure Children’s Mobile Device Use
Source: JMIR Form Res. 2022 Oct 21;6(10):e40452. doi: 10.2196/40452 (PMC9636534; doi:10.2196/40452)
Supplement: Multimedia Appendix 5 [file formative_v6i10e40452_app5.docx]

**Appendix 5. HealthSense versions tested and problems during study**

| **HealthSense Version** | **N** | **N**  **HealthSense worked on own device** | **Problem** | **N**  **Completed protocol** | **N**  **Unable to complete protocol** |
| --- | --- | --- | --- | --- | --- |
| **Feasibility Study A** | | | | | |
| Version 1  (Pop-up prompt after unlocking only) | 5 | 4 | **Description**: unable to connect to wi-fi with participant’s phone. Used study back up mobile phone (n=1) | 5 | 0 |
|  |  |  | **Reason**: unknown |  |  |
| **Feasibility Study B** | | | | | |
| Version 3  (Notification after unlocking) | 10 | 9 | **Description**: unable to install app. Used study back up mobile phone (n=1) | 10 | 0 |
|  |  |  | **Reason:** app required privacy updates |  |  |
| **Feasibility Study C** | | | | | |
| Version 1  (Pop-up prompt after unlocking only) | 3 | 1 | **Description**: user identification pop-ups did not appear (n=1)  Unable to install app (n=1) | 1 | 2 |
|  |  |  | **Reason**: unknown (OS version not collected) |  |  |
| Version 2  (notification after unlocking, 15 minute notification prompt, and + reminder) | 9 | 5 | **Description**: user identification notification did not appear (n=3).  App usage not recorded (n=1) | 5 | 4 |
|  |  |  | **Reason**: OS version (Android versions 5.0.2) did not support app (n=1).  Device model did not support app (n=3) |  |  |
| Version 3  (Notification after unlocking only, and reminder) | 9 | 7 | **Description**: app usage not recorded (n=2) | 7 | 2 |
|  |  |  | **Reason:** unknown |  |  |
| **Feasibility Study D** | | | | | |
| Version 3  (Notification after unlocking only, and reminder) | 12 | 7 | **Description**: option to allow usage tracking on tablet did not appear (n=1).  User identification notification did not appear (n=1).  Unable to install app (n=3) | 7 | 5 |
|  |  |  | **Reason**: older OS versions (Android versions: 4.4.2, 6.0, 6.0.1, and 7.1.1) |  |  |
